# A comprehensive review of the health benefits of flaxseed oil in relation to its chemical composition and comparison with other omega-3-rich oils

**DOI:** 10.1186/s40001-023-01203-6

**Published:** 2023-07-18

**Authors:** Somaia Al-Madhagy, Naglaa S. Ashmawy, Ayat Mamdouh, Omayma A. Eldahshan, Mohamed A. Farag

**Affiliations:** 1 Pharmacognosy Department, College of Pharmacy, Cairo University, Kasr El-Aini St., Cairo, 11562 Egypt; 2 Pharmacognosy Department, Faculty of Pharmacy, Ain Shams University, Cairo, 11566 Egypt; 3 Faculty of Pharmacy, Alexandria University, Alexandria, Egypt; 4 Present Address: Center of Drug Discovery Research and Development, Ain Shams University, Cairo, 11566 Egypt

**Keywords:** *Linum usitatissimum*, Flaxseed oil, Analysis, Omega 3, Health benefits, Lignans

## Abstract

Flaxseed (*Linum usitatissimum* L) is an ancient perennial plant species regarded as a multipurpose plant owing to its richness in omega-3 polyunsaturated fatty acids (PUFA) including α-linolenic acid (ALA). The extensive biochemical analysis of flaxseed resulted in the identification of its bioactive, i.e., lignans with potential application in the improvement of human health. Flaxseed oil, fibers, and lignans exert potential health benefits including reduction of cardiovascular disease, atherosclerosis, diabetes, cancer, arthritis, osteoporosis, and autoimmune and neurological disorders that have led to the diversification of flaxseed plant applications. This comprehensive review focuses on flaxseed oil as the major product of flaxseed with emphasis on the interrelationship between its chemical composition and biological effects. Effects reviewed include antioxidant, anti-inflammatory, antimicrobial, anticancer, antiulcer, anti-osteoporotic, cardioprotective, metabolic, and neuroprotective. This study provides an overview of flaxseed oil effects with the reported action mechanisms related to its phytochemical composition and in comparison, to other PUFA-rich oils. This study presents the most updated and comprehensive review summarizing flaxseed oil’s health benefits for the treatment of various diseases.

**Graphical Abstract:**

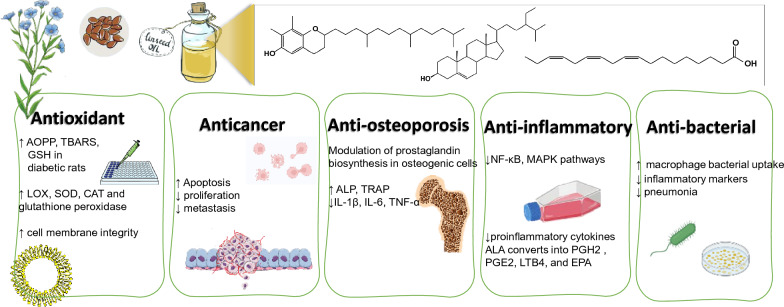

## Introduction

Flaxseed also called linseed (*Linum usitatissimum*) is a member of the genus Linum in the family Linaceae. Flaxseed has been used for centuries for everyday life as drying oil in painting, vanishing, and for medical purposes for respiratory disorders, constipation, abdominal pain, urinary tract infection, and skin inflammation [1]. Flaxseed typically yields 35–45% oil, containing 9–10% of saturated fatty acids (palmitic and stearic), about 20% monounsaturated fatty acids (mainly oleic acid), and more than 70% α-linolenic acid. Flaxseed contains a protein content ranging from 20 to 30%, while its dietary fiber content can reach as high as 28% [2]. The flaxseed oil components differ according to their seed cultivar, location, environmental condition, and methods of analysis [3]. Flaxseed oil is well recognized for its health benefits attributed to its unique chemical composition presenting one of the richest sources of polyunsaturated omega-3 fatty acids from plant sources. Flaxseed oil is rich particularly in linoleic and α-linolenic fatty acids as its main constituents [4]. Linoleic acid (LA; 18:2n26) and α-linolenic acid (ALA; 18:3n-3) are metabolized upon ingestion by desaturation and elongation to 20 carbon fatty acids. Linoleic acid (n-6) is converted to arachidonic acid, whereas α-linolenic acid (n-3) is converted to eicosapentaenoic acid (EPA). Arachidonic acid is the progenitor of PGH2 that is further converted to PGE2 and TXA2 via a cyclooxygenase. Arachidonic acid also serves as a precursor of LTA4 that will be converted into LTB4 by using 5-lipoxygenase [5]. EPA is an n-3 homologue that can inhibit arachidonic acid metabolism via competitive inhibition. EPA serves likewise as a substrate for 5-lipoxygenase which leads to the formation of LTB5 to exert mild inflammatory activity compared with LTB4 [6]. The α-linolenic acid is widely used as an anti-inflammatory agent by decreasing the production of inflammatory cytokines, lipids, and lipoprotein [7]. Inhibition of proinflammatory cytokines production provides further action on bone reabsorption by upregulating the proliferation and activation of the osteoclast in addition to stimulating osteoblast to release osteoclast differentiation factor [8]. The linolenic acid also exerts antimicrobial effects that functions via the inhibition of bacterial enoyl-acyl carrier protein reductase [9]. The beneficial health effects of flaxseed oil include reduction in the risk of cancer and cardiovascular diseases as well as decreasing cholesterol levels [10]. Flaxseed oil also exhibit an antioxidant activity. Such antioxidant activity depends on the quantity of oil chemical components. Flaxseed oil is rich in several antioxidants like tocopherols, beta-carotene [11], phytosterols, polyphenols, and flavonoids [12]. Tocopherols are group of important lipid-soluble phytocompounds that are mainly present in four isomers, *α, β, γ*- and *δ*, and have multiple physiological activities, including antioxidant activity and anticancer effects [13]. The antioxidant activity of tocopherols is mainly due to their ability to donate their phenolic hydrogens to lipid free radicals. The strength of the antioxidant activity of the tocopherols in vivo was found to be in the following order *α* > *β *> *γ* > *δ* [14]. Whether a synergized effect occurs with tocopherols and omega fatty acids in flaxseed oil with regard to antioxidant action has yet to be determined.

In a study that examined the effect of different extraction methods on flaxseed oil composition and in relation to its antioxidant activity, only three kinds of tocopherol isomers *α*, *γ*, and *δ* were detected. It was found that hexane-extracted oil encompassed higher levels of tocopherols 614 mg/kg compared with hot pressed oil at 160 °C (483 mg/kg, respectively), and cold pressed oil (564 mg/kg). Moreover, roasting process significantly affected tocopherols level in flaxseed oil. Cold pressed oil encompassed 12.2, 43.4, and 508 mg/kg of α, γ, and δ-tocopherol. Although, γ-tocopherol was the most abundant type in all extraction methods, it was suggested that best extraction conditions of α- and γ-tocopherols was at a temperature that did not exceed 120 °C (13.0 and 553 mg/kg, respectively). Meanwhile, δ-tocopherol content dropped to reach 43.1 mg/kg at such temperature. Interestingly, upon increase of roasting temperature to 160 °C, a significant drop in α- and γ-tocopherol content (11.3 and 472 mg/kg) was reported, whereas *δ*-tocopherol was not detected [12]. Extraction optimization for flaxseed oil targeting other health effects should now follow for the identification of best extraction methods.

With regard to phytosterols composition, 6 kinds of phytosterols were detected in flaxseed oil. β-Sitosterol was the dominant isomer, followed by cycloartenol, campesterol, Δ5-avenasterol, 2,4-methylenecycloartenol and stigmasterol. The cold pressed oil contained the highest content of total phytosterols (11.8 g/kg) which showed a decreasing trend when oil was extracted from roasted seeds at 120 °C and 160 °C (11.3 and 9.65 g/kg).

**Fig. 1:**
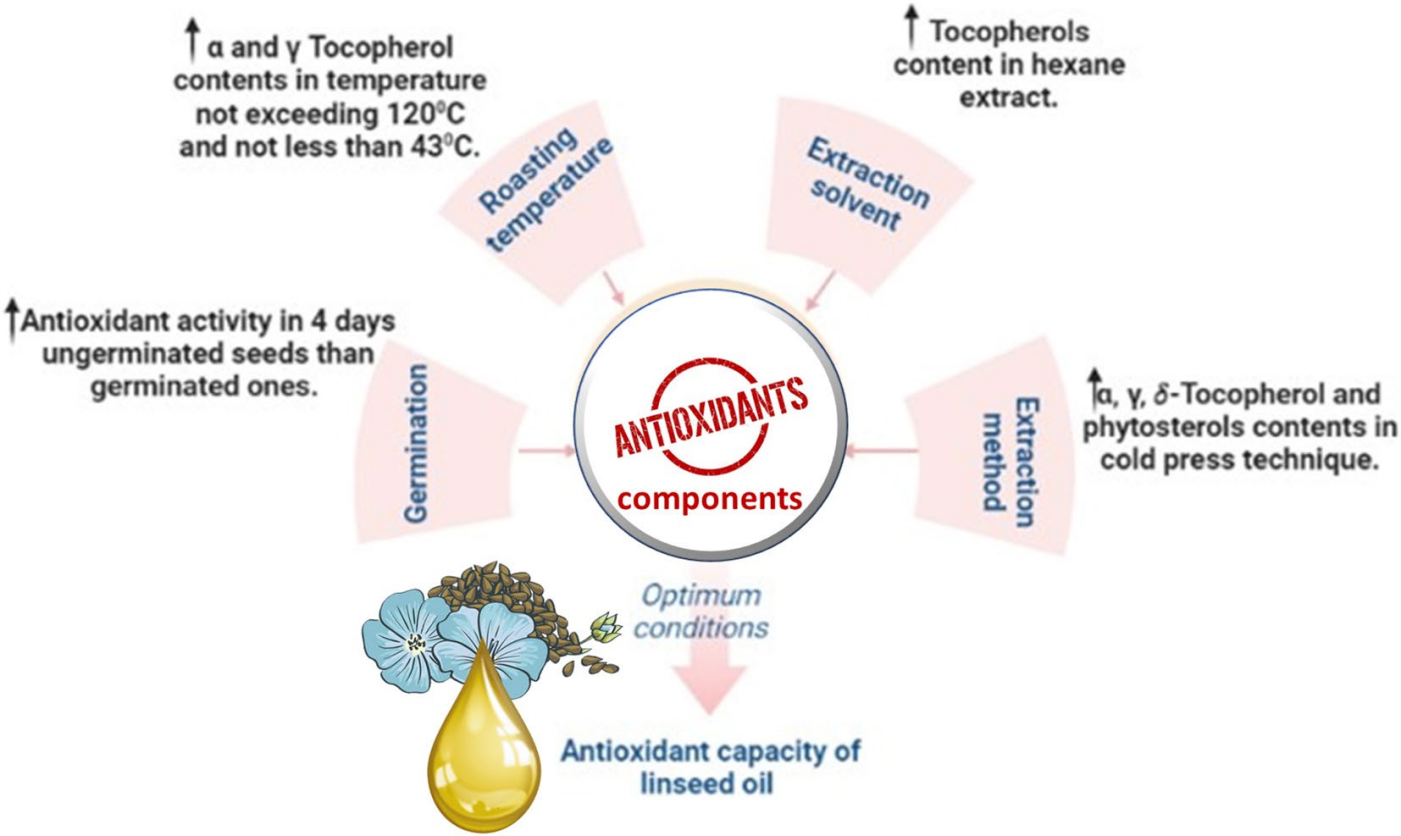
Diagrammatic sketch showing factors affecting flaxseed antioxidant effect including; extraction method, extraction time, seed roasting and germination

The concentration of Δ5-avenasterol, 2,4-methylenecycloartenol and stigmasterol were not affected largely by cold pressed, hot 120 °C and hot 160 °C methods but their concentration reasonably increased using solvent extraction method (Fig. 1) [12].

**Fig. 2:**
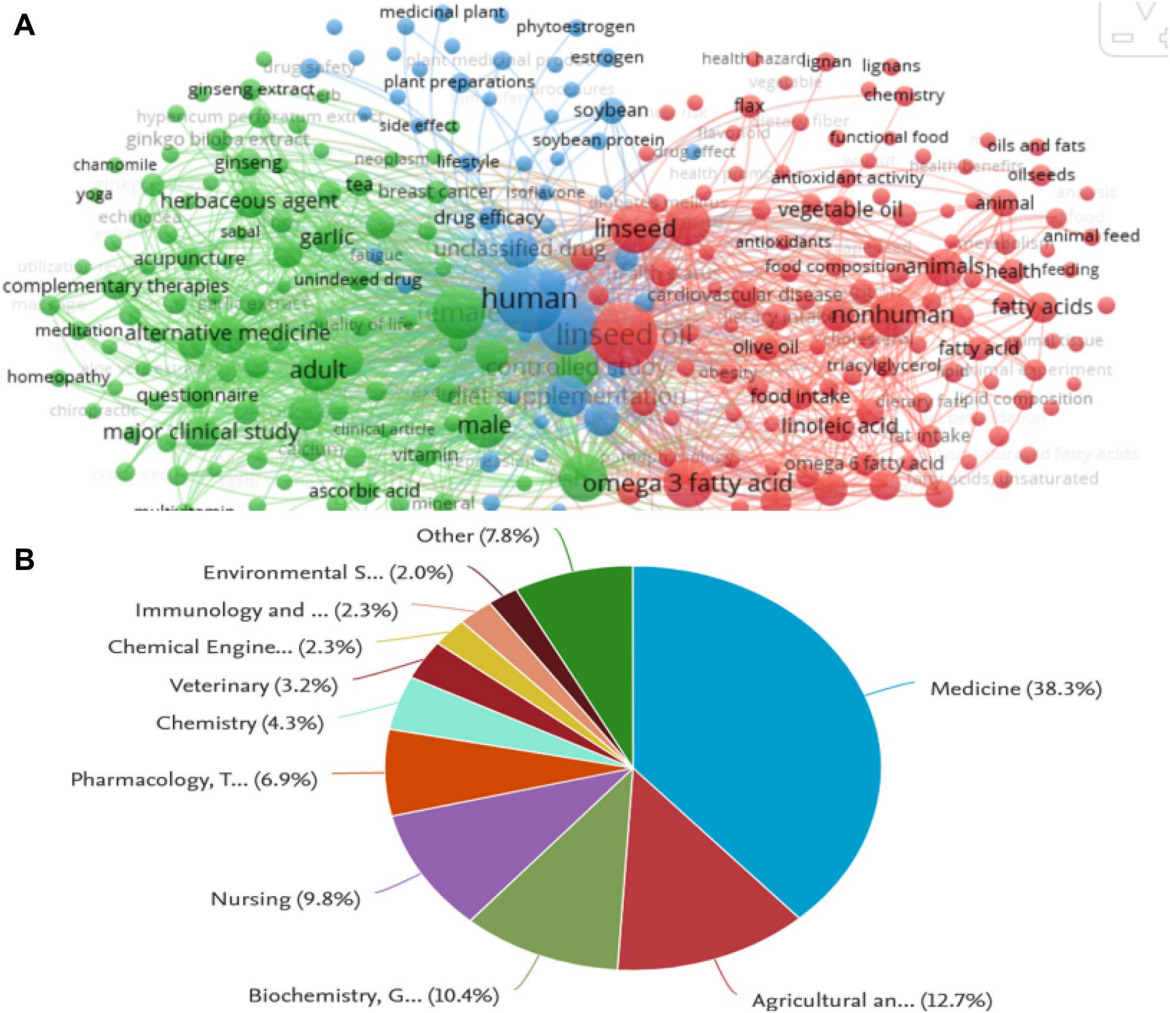
**A** Analysis of relevant keywords of flaxseed oil uses research during 2001–2022 from Scopus (collected on 20th October 2022). Irrelevant literature was removed by manual inspection and visualized using VOS viewer. **B** Pie chart showing paper retrieved by subject area by searching Scopus database for the term flaxseed and uses from 2000 till now

Moreover, fatty acid from flaxseed contributes to various metabolic processes of the cell, including cell membrane structural components and storage lipids [15]. The hypocholesterolemic effect of flaxseed oil is attributed to several chemicals including omega-3 fatty acids in addition to lignans which exert both hypolipidemic and antioxidant effects [16, 17]. Such a rich yet under-exploited oil resource, has been the subject of research from various viewpoints over the last 20 years due to its several health benefits (Fig. 2A). Papers grouped by subject areas revealed that 38% belonged to medicine among all categories and highlighting for its potential health benefits (Fig. 2B).

**Fig. 3:**
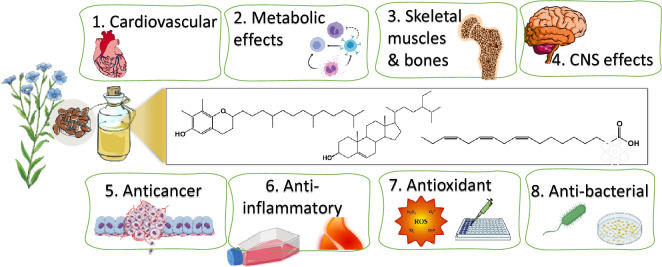
An outline showing the review theme and covered topics related to flaxseed oil biological effects and its chemical composition

The main goal of review is to summarize health benefits of flaxseed oil from those reported from whole seeds in relation to its chemical composition. The effects discussed, as evidenced by cell-based assays, animal assays, and clinical trials, encompass a wide range of properties including antioxidant, anti-inflammatory, antimicrobial, anticancer, antiulcer, anti-osteoporotic, cardioprotective, and neuroprotective effects. Figure 3 provides an outline of the review theme and covered effects.

## Methods

Herein, we aimed at conducting a comprehensive literature review on flaxseed oil health benefits. An extensive search was performed using databases including Web of Science, PubMed and Scopus using key terms including flaxseed oil as one search item and targeted biological effects viz. antioxidant, anti-inflammatory, antimicrobial, anticancer, antiulcer, anti-osteoporotic, cardioprotective, neuroprotective and metabolic syndrome. The inclusion criteria mainly focused on a defined proven role of flaxseed oil in health benefits. Owing to discrepant findings across primary studies and the significant high number of articles, each citation was independently screened using title and abstract. Only the relevant citations, original researches then underwent a second stage of full-text screening. Exclusion criteria included papers preprints, conference proceedings, papers for which only abstract was found or papers not published in English. Finally, the reference lists of retrieved key papers were also examined for articles of relevance to reach 112 papers cited in the text.

### Flaxseed oil health benefits

#### Risk against the heart

The positive effect of polyunsaturated omega-3 fatty acids in mitigating against the risk of heart diseases is well reported in literature [18–20]. Studies revealed that both eicosahexaenoic acid (EPA, 20: 5n-3) and docosahexaenoic acid (DHA, 22: 6n-3), the main functional constituents of cold fish oil, are associated with lowering risk of cardiovascular diseases such as arrhythmia [21, 22], sudden cardiac death [23] and atherosclerosis [24]. Flaxseed oil is rich in α-linolenic acid which is considered a natural precursor of EPA and DHA. Both EPA and DHA in the erythrocyte membrane increase in response to supplementation with fish oil and flaxseed oil at rates related to the amount of the supplement. Supplementation with flaxseed oil increased EPA in the erythrocyte membrane to 133% (*P* < 0.05) and increased docosapentaenoic acid (DPA, 22:5 3) to 120% (*P* < 0.01) of the baseline level, however, DHA remained unchanged [25]; whereas supplementation with cold fish oil increased both EPA and DHA in the erythrocyte membrane to 300% (*P* < 0.001) and 42% (*P* < 0.001), respectively [25]. The cardiovascular protective effect of flaxseed oil is attributed mainly to its lipid lowering as well as its antioxidant effects [16, 17]. Comparison between flaxseed oil and other oils rich in omega-3 fatty acids regarding production rate of EPA and DHA in erythrocyte membrane has yet to be reported in literature. Further, whether synergistic effect exists upon administration of flaxseed oil and classical cardiovascular drugs has yet to be reported.

#### Metabolic effects

The role of omega-3 fatty acids in reducing the risk of cardiac arrhythmia and sudden death in coronary heart disease patients is well reported. Moreover, omega-3 fatty acids are also useful in the treatment of hyperlipidemia and hypertension [26]. α-Linolenic acid from flaxseed oil is well reported for its hypocholesterolemic effect [27].

A study investigated the effect of flaxseed oil on high-cholesterol diet-induced blood lipids, atherosclerosis, serum malondialdehyde, aortic chemiluminescence, and reactive oxygen species (ROS) in rabbits. Results indicated that the levels of serum total cholesterol, low-density lipoprotein cholesterol, high-density lipoprotein cholesterol, triglycerides, as well as serum malondialdehyde and antioxidant markers, were similarly increased in both groups: the 0.5% cholesterol diet group and the group consuming a diet containing 0.5% cholesterol and 5% flaxseed oil. These increases were observed in comparison to the control group and the group consuming 0.5% flaxseed. White blood cells’ ROS-producing activity was elevated in 0.5% cholesterol diet group, and flaxseed oil prevented such increase induced by cholesterol [28]. These results were confirmed by other studies reporting the improvement of plasma lipid profile and in tissue lipids composition [29–31].

Furthermore, a clinical study assessed the effects of oils rich in ALA such as flaxseed and fish oil enriched diet on plasma lipids and low-density lipoproteins (LDL) compared to sunflower oil. This study involved 57 men having an atherogenic lipoprotein phenotype (ALP) that were assigned randomly to one of three diet regimens enriched either with flaxseed oil (FXO: high ALA, *n* = 21), sunflower oil (SOF: high linoleic acid, *n* = 17), or fish oil (SO, *n* = 19) administered for 12 weeks, reaching dietary consumption ratios of C-6: C-3 PUFA of 0.5, 27.9 and 5.2, respectively. Results showed that ALA and EPA relative abundance in erythrocyte membranes increased in flaxseed oil diet (*P* < 0.001), while EPA and DHA increased following fish oil (*P* < 0.001). Total plasma cholesterol showed reduction within (FXO -12.3%, *P* = 0.001; SOF − 7.6%, *P* = 0.014; SO − 7.3%, *P* = 0.033) with FXO showing the strongest effect [32].

In another clinical study comparing the effects of two oils rich in essential fatty acids that are hempseed oil and flaxseed oil on serum lipids, plasma glucose and insulin, and hemostatic factors in healthy 14 human subjects. A randomized, double-blind crossover study design was applied and the healthy volunteers consumed both oils (30 ml/day) for 4 weeks. Higher proportions of both linoleic acid and gamma-linolenic acid in serum cholesteryl esters (CE) and triglycerides (TG) was observed in hempseed oil compared with flaxseed oil (*P* < 0.001), while flaxseed oil showed a higher proportion of ALA in serum CE and TG compared with hempseed oil (*P* < 0.001) [33].

ALA from flaxseed oil was further reported to exert a beneficial effect in the treatment of obesity and improvement of insulin resistance [34, 35] as well as in protection against diabetic retinopathy [36]. Supplementation with conjugated linoleic acid reduced C-6 and C-3 weight% liver lipids level in mice by 57 and 73% concurrent with increase in C-6: C-3 ratio by 58% compared to control group. Flaxseed oil increased both C-6 and C-3 fatty acids in mice liver lipids by 33 and 342% and reduced C-6:C-3 ratio by 70% [34]. Moreover, flaxseed oil was reported to enhance plasma adiponectin levels which is released from the adipose tissues to improve insulin sensitivity. In this study, rats were fed safflower oil and flaxseed oil for 4 weeks with plasma adiponectin concentration in the flaxseed oil-fed group, detected at 1.5 fold higher level than that in the safflower fed group [37] and posing flaxseed oil as healthier alternative than safflower.

Prospective, parallel-arm design involving two groups was conducted to evaluate the effect of dietary supplementation with flaxseed oil (8 g/day) on blood pressure in middle-aged dyslipidemic 59 men for 12 weeks. Blood pressure was measured at the beginning of the dietary intervention period as well as at the end. Group supplemented with flaxseed oil showed significant decrease in both systolic and diastolic blood pressure levels compared with the control group (*P* = 0.016 and *P* = 0.011, respectively [38]. The antihypertensive action mechanism of flaxseed oil has to be fully elucidated using rat animal or ideally clinical trials.

As a conclusion, flaxseed oil consumption can decrease the risk of cardiovascular diseases such as cardiac arrhythmia in addition to its benefit to hypertensive and hyperlipidemic patients. The hypocholesterolemic effect of flaxseed oil, and its ability to improve lipid profile is attributed mainly for its richness in α-linolenic acid. So far, further research studies are still needed to fully understand the underlying action mechanisms behind the antihypertensive effect of flaxseed oil. According to the reported data, adding flaxseed oil to diet appear to be useful to reduce the risk of cardiovascular disease.

#### Effects on skeletal muscle and bone health

In skeletal muscle, the movement of lipids across the sarcolemmal membrane is regarded as the rate-limiting step in fatty acids oxidation. Consumption of flaxseed oil high in α-linolenic acid affects plasma membrane lipid composition and the capacity to transport palmitate. Rats fed a diet supplemented with α-linolenic acid (10%) showed an increase in omega-3 polyunsaturated fatty acids (PUFAs) in the whole muscle and sarcolemmal membranes (fivefold). Moreover, increase in sarcolemmal palmitate transport rates (20%), rates of whole body fat oxidation (~ 50%), and skeletal muscle triacylglycerol content (two fold) were observed [39].

Diets rich in PUFA as in the form of flaxseed oil, were found to exert a beneficial effect on femur bone mineral density, bone strength, and fatty acid composition. Femurs of male rats (6 to 15 weeks of age) fed high n-3 PUFA diets were found stronger than the chow-fed group of rats [40]. On the other hand, the effect of flaxseed oil on bone damage induced by a high-fat diet was assessed in rats revealing that flaxseed oil ameliorated trabecular bone damage and promoted osteoblastic function as well as promoted osteogenesis [41].

Another animal study suggested that consumption of a variety of n-3 PUFA sources to promote bone health during the growth stage. Rats fed with flaxseed oil rich in alpha-linolenic acid showed improvement in the bone microarchitecture compared with rats fed with salmon oil. Serum osteocalcin was observed to be higher (*P* = 0.03) in flaxseed-fed rats, with osteocalcin likely to be related to improved trabecular bone microarchitecture [42].

Conclusively, the consumption of flaxseed oil can improve skeletal muscle lipid composition and promote bone health. Diets high in C-3 polyunsaturated fatty acids have been shown to improve femur bone mineral density and bone strength. Flaxseed oil has also been found to ameliorate trabecular bone damage. While further studies are still required to compare the efficacy of flaxseed oil versus other omega-3 rich oils, it is clear that flaxseed oil can promote bone health through various mechanisms but mainly due to enhancement of osteogenesis.

#### Anti-osteoporotic activity

Osteoporosis is a metabolic bone disorder characterized by low bone density and deformation in bone construction that often leads to an increased risk of fractures [43]. According to WHO, the global prevalence of osteoporosis is estimated to be 19.7% [44]. During lifespan, bones are constantly resorbed by osteoclasts and replaced with new ones by osteoblasts. This process maintains bone mechanical strength and repair. Any imbalance in this remodeling process in the form of increased bone resorption at the expense of bone formation could result in osteoporosis [45]. Bone formation is regulated by many factors including hormones. Estrogen and testosterone have a substantial influence on bone remodeling mainly by inhibiting bone breakdown [46]. Cytokines and prostaglandins can also influence bone remodeling by decreasing bone resorption [47].

To understand the osteoprotective effect of flaxseed oil, one should have a look at the whole process. Whole flaxseed contains two phytochemical components capable of modifying bone metabolism that are its oil and lignan fractions. Flaxseed oil is rich in α-linolenic ALA to serve as metabolic precursor of eicosapentaenoic acid (EPA) and docosahexaenoic acid (DHA). These acids play a major role in modulating osteoblastogenesis, osteoclastogenesis, inflammatory processes, eicosanoid production, and calcium metabolism [48]. On the other hand, flaxseed lignans are phytoestrogens that exert estrogenic/antiestrogenic effects on the estrogen receptor. Although secoisolariciresinol diglucoside (SDG), the main lignan component in flaxseed, has also been detected in flaxseed oil (4 mg/kg) [49], almost all studies attributed the biological effect of flaxseed oil to its PUFAs content rather than SDG.

**Fig. 4:**
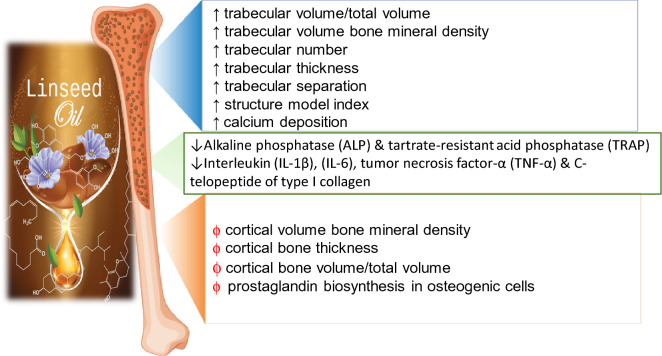
Diagrammatic sketch showing the anti-osteoporosis mechanisms of flaxseed oil and its components. Blue box; illustrates the trabecular part of bone, red box; shows the cortical part of bone. Upwards arrow indicates marker improvement. Downwards arrow indicates marker decrease. Red Phi symbol indicates no significant change in the osteoporosis marker

To shed more light on the component responsible for the beneficial effect of flaxseed on bone health, a study showed that the consumption of whole flaxseed did not lead to a meaningful improvement of bone mass in humans and animals. However, when flaxseed was combined with estrogen regimen, it exerted an extra benefit on the bone in animals [50]. Similar findings were found for flaxseed oil, but more favorable results were reported when flaxseed oil was consumed in the presence of other pathological conditions such as kidney disease [50–52] In contrast, the consumption of flaxseed lignans resulted in a less positive effect on bone, with effect found limited to the early life of female animals only [50]. These findings suggest that flaxseed oil is more effective than flaxseed lignans, and whether comparison of flaxseed oil to whole seeds in osteoporosis models yield superior effect for oil versus whole seed has yet to be reported. A summary of flaxseed oil anti-osteoporosis effect is presented in Fig. 4.

Recently, increasing evidence points to that ingestion of ω-3 polyunsaturated fatty acids (PUFAs) such as ALA, the main component of flaxseed oil, improves osteoporotic status [53, 54] through modulation of prostaglandin biosynthesis in osteogenic cells [55]. A study aimed to examine the role of feeding ovariectomized rats by 10% flaxseed oil for 4 weeks showed significant decrease in the activity of osteoblast differentiation marker, Alkaline phosphatase (ALP), and osteoclast marker, Tartrate-resistant acid phosphatase (TRAP), from 300 ± 49.1 and 0.80 ± 0.08 in control group to 166.80 ± 17.9 and 0.37 ± 0.08 in treatment group, respectively. In addition, histopathological examination of distal femur showed thick elongated tubercula and narrow inter-tubercular space in treatment group [56]. It has been found that fatty diet rich in n-3 fatty acids can also reduce levels of serum cytokines such as interleukin-1β (IL-1β), interleukin-6 (IL-6), and tumor necrosis factor-α (TNF-α). These cytokines exert a crucial role in bone resorption either by activating the proliferation of the osteoclast or by stimulating osteoblast to release osteoclast differentiation factor [57]. The effect of consuming flaxseed oil on bone metabolism during growth is though still controversial. Some studies showed that the consumption of a 10% ground flaxseed diet (as a source of flaxseed oil and lignans) to newborn rats caused a reduction in their bone strength compared to the control group fed with purified flaxseed lignan alone. Researchers attributed this harmful effect to flaxseed oil, rather than its lignan component [58]. In contrast, other studies showed that flaxseed oil does not affect piglets’ and mice bone mass [8, 59], while others proved that flaxseed oil provides a protective effect against ovariectomized (OVX) induced alveolar bone loss better than that of menhaden oil, fish oil containing the metabolic products of ALA, EPA and DHA [60]. Regarding cod liver oil, the other major source of ω-3 PUFAs, it has been found that OVX rats fed by 200 ml/kg body weight daily of cod liver oil for 8 weeks showed significantly higher levels of calcium deposition estimated by plasma calcium and femur calcium levels compared to both sham and OVX control groups (9.3 ± 0.9, 10.2 ± 0.6, 6 ± 2.1 mg/dl, respectively) and (93 ± 14, 105 ± 21 and 60 ± 11 mg/g, respectively) as well as a significantly lower bone turnover, as indicated by level of osteocalcin compared to the same groups (69 ± 6.1, 98.6 ± 9.3, 65 ± 5.9 ng/ml, respectively). Noteworthy, cod liver fed rats results were close to plasma calcium, femur calcium and osteocalcin levels in estrogen fed rats (9.8 ± 0.5 mg/dl, 95 ± 10 mg/g, 70 ± 7.3 ng/ml, respectively) [61]. Osteoporosis was hypothesized to be a feminine disease. However, a recent study revealed that abdomen obesity in males can signal osteoporosis [62]. A detailed study was conducted to examine the ameliorative effect of consuming high-fat diet containing 60% fats and 10% flaxseed oil (HY diet) for 22 weeks against high fat diet-induced bone damage in rats. The study measured different biological and histopathological parameters to evaluate bone microstructure and biomechanics. Results, surprisingly, revealed two opposite effects on the level of trabecular and cortical bone markers in rats receiving HY diet (HY group) compared to control groups that were fed a normal diet (NC group) or diet containing 60% high-fats alone (HFD group). All trabecular markers including bone volume/total volume, trabecular volume bone mineral density, trabecular number, trabecular thickness, trabecular separation and structure model index (SMI) were improved. However, cortical volume bones mineral density, cortical bone thickness (Ct. Th), and cortical bone volume/total volume were not significantly different among all groups. Furthermore, serum markers reflecting bone formation, ALP, and total procollagen type 1 N-terminal propeptide, were found higher in the HY group, while serum cross-linked C-telopeptide of type I collagen, the marker reflecting bone resorption, was lower in the HY group compared with HFD group. In addition, it was found that treatment of primary osteoblasts with (1, 10 and 100 µM) ALA, major component in flaxseed oil, significantly restored palmitic acid-induced inhibition of *β*-catenin, RUNX2, and osterix proteins in a dose-dependent manner [41].

#### Effects on central nervous system and depression

Pre-treatment with flaxseed oil in a rat model of brain ischemia revealed for a potential neuroprotective effect on the motor cortex area following cerebral ischemic stroke. This effect was due to increase in the neurotrophic factors; brain-derived neurotrophic factor and glial cell-derived neurotrophic factor [63]. Similarly, another study where rats were fed flaxseed oil in microemulsion, the brain synaptic membrane were enriched with DHA leading to enhanced dopamine and serotonin levels inside the brain [64]. How would flaxseed oil synergize antidepressant drugs targeting improved serotonin levels should now follow?

The fish oil omega-3 fatty acids DHA and EPA, are essential components of cell membranes and are likewise critical for brain functions [65]. It has been reported that omega-3 fatty acids were involved in many mood disorders [66]. Epidemiological studies revealed that the consumption of fish is inversely related to depression [67]. Similarly, a dietary supplement of flaxseed oil exerted a promising effect in the treatment of depression. It has been shown that both behavioral despair and anhedonia were improved by feeding 10% flaxseed oil in rats exposed to unpredicted chronic stress [68].

Moreover, an Egyptian cultivar of flaxseed oil was reported to exhibit significant antidepressant-like activity in a rat model of postpartum depression. Oral administration of flaxseed oil (270 mg/kg/day) for 2 weeks during the postpartum period improved anxiety and depressive symptoms in postpartum depression-induced rats as revealed by assessment of anxiety-like behaviors (improved plus maze, forced swim test tests, and open field test) [69]. Comparison of flaxseed oil prepared from different cultivars or other origin regarding their CNS effects compared to chemical composition shall aid identify best sources of that seed for oil production.

#### Anticancer activity

Natural compounds are a rich source for antioxidants [70] and anticancer agents as well reported from plant sources [71]. Several studies reported the cytotoxic effect of flaxseed oil against various types of cancer cells [72–74]. Omega-3 polyunsaturated fatty acid, α-linolenic acid, as well as its metabolites, EPA and DHA have been reported to in vitro inhibit breast cancer cell growth [75].

Flaxseed oil reduced the growth of a murine cell line of melanoma B16-BL6 in a dose-dependent manner. Treatment of B16-BL6 melanoma cells with 0.3% (v/v) as a low dose of flaxseed oil for 4 days reduced the number of cells by *ca*. 50%, while treatment with 0.9% (v/v) as a high dose completely inhibited cells growth. Similarly, other malignant cell lines showed growth inhibition effect with 0.3% (v/v) flaxseed oil including a decrease by 25% for HeLa, 75% for MCF-7, and 50% for both MDA-MB-231 and MDA-MB-468 cells. Higher growth inhibition was observed at higher dose of 0.9% oil. Moreover, MTT viability assay showed that the growth of cancer cells was inhibited by 40–60% after 4 days of treatment with flaxseed oil, while non-malignant cells were not affected suggestive for its safety compared to chemotherapeutics. Furthermore, treatment with flaxseed oil disrupted mitochondrial function in both MCF-7 and B16-BL6 cells [76], and whether other mechanisms mediate for flaxseed oil cytotoxic effect should be examined in details.

With regard to the identification of active antitumor agent in flaxseed oil, linusorbs as natural peptides occurring in flaxseed oil exerted cytotoxic effect on glioblastoma cells. Linusorbs induced apoptosis and inhibited proliferation of both glioblastoma and breast cancer cell lines. Glioblastoma C6 cells were treated with several doses of linusorbs for 48 h, and cell viability was tested using an MTT assay. Linusorbs at a dose of 20 to 40 µM exhibited cytotoxicity in a dose-dependent manner, while at doses lower than 10 µM no cytotoxic effect was observed. C6 Cells treated at a dose of 20 µM were then cultured for 72 h, with significant decrease in cell proliferation rate compared to untreated control cells. Similarly, a significant reduction of viability of the other glioblastoma U251 cells at doses of 20 µM whereas, doses lower than 10 µM showed no effect. In contrast, breast cancer MDA-MB-231 cells were found more sensitive to linusorbs found to exert a cytotoxic effect at a dose of 10 µM, compared with both glioblastoma cell lines that showed sensitivity at 20 µM and suggestive that drug sensitivity and cytotoxicity depend on cancer cell type [77], which has yet to be confirmed using tumor grafted animals to be conclusive for flaxseed oil.

More studies investigated the cytotoxic effect of flaxseed oil especially against breast cancer among other types. The effect of flaxseed oil on the growth of human breast cancer (MCF-7) in ovariectomized mice at high premenopausal-like estrogen levels was evaluated. Mice with established MCF-7 tumor were either fed a basal diet (as a control group) or a basal diet with flaxseed oil (40 g/kg) for 8 weeks. In comparison with the control group, flaxseed oil decreased tumor size (33%, *P* < 0.05) as well as tumor cell proliferation (38%, *P* < 0.05) as well as inducing apoptosis (110%, *P* < 0.001) [78]. It was also reported that flaxseed oil reduced metastasis after the surgical excision of solid human breast tumor in mice [79].

In another study in athymic mice, the anticancer effect of combined low dose trastuzumab treatment (2.5 mg/kg body weight) with 8% flaxseed oil was compared to the effect of the combined high dose trastuzumab treatment (5 mg/kg body weight) with 8% flaxseed oil on (HER2) positive breast cancer cells. Results showed that control tumors grew 187%, while combined flaxseed oil and low dose trastuzumab showed 84% regression after 4 weeks of treatment. Flaxseed oil synergized the tumor reduction effects of trastuzumab and the combined flaxseed oil and low dose trastuzumab showed similar efficacy to the high dose treatment of trastuzumab [80] to aid in lowering the dose of chemotherapeutics for cancer treatment.

Likewise, cytotoxicity of flaxseed oil and its combination with another chemotherapeutic that is doxorubicin (Dox) was evaluated against MCF-7 and MDA-MB231 cell lines using SRB assay. Treatment of both MCF-7 and MDA-MB231 cell lines with flaxseed oil phenolic extract alone showed growth inhibition with IC_50_ value of. 63 µg/ml, versus an IC_50_ value of 1.2–1.5 µg/ml with single Dox treatment. On the other hand, the antiproliferative effects of Dox and flaxseed oil phenolic extract combinations were evaluated by testing equipotent doses of the two agents at a ratio, of 50:50. Synergistic effect was quantified by measuring combination index (CI) [81] where CI < 0.9 indicates synergistic effect.

Results revealed that combining Dox and flaxseed oil phenolic extract (50:50) led to a reduction in IC_50_ values to reach 15.7 µg/ml (flaxseed oil extract) and 0.3 µM (Dox) for MCF-7 cells. Moreover, a significant synergistic effect with CIs (CIs < 0.9) was demonstrated upon cell line treatment with equipotent combination doses. After combined treatment a dose decrease by 20-fold for Dox and 7.7-fold for flaxseed oil extract against MCF-7 cells and similar response was observed in MDA-MB231 cells when compared to the individual doses of each treatment [82].

In a similar study, the combined effect of secoisolariciresinol diglucoside (1 g/kg) and flaxseed oil (38.5 g/kg) was studied on the growth of human estrogen receptor-positive breast tumors (ER+) MCF-7 over a period of 8-week treatment. Application of 2 × 2-factorial analysis to determine the interaction effect of secoisolariciresinol diglucoside and flaxseed oil at *P* < 0.05 has been performed. Results revealed a significant interaction (with *P* = 0.038) between secoisolariciresinol diglucoside and flaxseed oil on the proliferation of MCF-7 cells compared to control. Secoisolariciresinol diglucoside decreased MCF-7 cell proliferation by 25.9% (*P* = 0.007), whereas flaxseed oil showed 22.9% reduction (*P* = 0.015). The interaction of both agents on cell apoptosis was not though considered significant [83].

The chemopreventive effect of flaxseed oil on carcinogenesis in rats was also investigated [84–86] against colon cancer. Colon tumor incidence, size, and multiplicity were reported to be at 82.6% and 29.4%; 44.4 and 5.3 mm, and 1.3 and 0.3 in corn and flaxseed meal groups, respectively. Colon and blood samples of the flaxseed meal group showed higher levels of omega-3 fatty acids. Moreover, COX-1 and COX-2 expression in this group were significantly lower (*P* < 0.05) compared to the corn group [84]. Another study reported that post-initiation dietary administration of flaxseed oil suppressed dimethylhydrazine induced colon carcinogenesis in rats without significant side effects [85].

#### Anti-inflammatory effect

Anti-inflammatory agents are widely derived from natural sources [87]. Inflammation is a defense mechanism that the body undertakes to kill any foreign body and to repair the damaged tissue to restore the homeostasis in the injured area. Inflammation is characterized by pain, redness, and swelling and in case of severe inflammation, it will be accompanied by complete loss of function. The inflammatory response is a self-limiting process that is regulated to not cause excessive to the host and all of that is by negative feedback mechanisms [88]. The pathology of inflammation can be explained by [1] an increased blood supply to the site of inflammation, [2] increased capillary permeability, [3] leukocyte migration from the capillaries into the surrounding tissue, [4] releasing some inflammatory mediators from leukocytes at the inflammation site and these inflammatory mediators are lipid mediators (e.g., n-6 eicosanoids, prostaglandin E2 (PGE2), and leukotriene B4 (LTB4) [89]. There are also other important peptide mediators (e.g., cytokines), interleukin 1b (IL-1b), and tumor necrosis factor-a (TNF-a), reactive oxygen species (superoxide), amino acid derivative (histamine), and enzymes (matrix protease) depending upon the cell type involved [88]. EPA is also a cyclooxygenase substrate for the biosynthesis of PGE3 which also exert inflammatory activity, albeit PGE3 biosynthesis occurs with very low efficiency [5]. EPA likewise acts as precursor for the production of prostaglandins with three double bonds and leukotrienes with five double bonds. These altered products are believed to mediate for the anti-inflammatory effects of ALA [90] and DHA can also yield resolvins and other related compounds (e.g., protectins) through pathways involving cyclooxygenase and lipoxygenases [91]. These mediators act synergistically as anti-inflammatory, i.e., resolvin E1, resolvin D1, and protectin D1 to all inhibit transendothelial migration of neutrophils preventing their infiltration at sites of inflammation. Further, resolvin D1 inhibits IL-1β production, whereas protectin D1 inhibits TNF and IL-1β production [91].

#### Antimicrobial and immunomodulatory effects

Natural products are well recognized for their potential antimicrobial effects [92–94]. Fish oil was reported for its anti-inflammatory effect, and in the management of other inflammatory disorders like rheumatoid arthritis [95]. Similarly, flaxseed oil was assessed for its anti-inflammatory, immunoregulatory and antibacterial effects mostly related to its richness in n-3 PUFAs [96–98].

It was reported that increasing flaxseed oil proportion in human diet can reduce immune function markers [98]. Amelioration of acute pneumonia was reported to be achieved by increasing dietary supplements with flaxseed oil polyunsaturated fatty acids [99]. The effect of flaxseed oil supplementation on the course of pneumonia caused by *Streptococcus pneumoniae* in a mice model was studied. Results revealed that long-term supplementation protected animals against lungs’ bacterial colonization with *S. pneumoniae* with decreased histopathological involvement of lung tissue. Moderate pneumonia was detected in supplemented infected mice in comparison to severe pneumonia in control mice. Moreover, a decrease in inflammatory markers was observed. On the other hand, short-term supplementation did not show an effect on lung colonization [97].

To assess the role of n-3 PUFA on apoptosis and the process of macrophage phagocytic activity in mice, three groups of mice (*n* = 60) were compared; first group was fed on cod oil, while the second group was fed on flaxseed oil and a third control group was fed on a standard diet. After 9 weeks of supplementation, apoptotic and phagocytic activities of alveolar macrophages were evaluated. Significant increase in both bacterial uptake and intracellular killing (*P* < 0.05) of *S. pneumoniae* was observed in the alveolar macrophages from sea cod oil and flaxseed oil-fed groups. Moreover, alveolar macrophages of these groups showed a significant reduction (*P* < 0.05) in apoptotic cells, while maximum apoptosis was shown in alveolar macrophages of control group on interaction with the bacteria. These results confirmed that dietary supplementation of PUFA can enhance phagocytic capability of alveolar macrophages and reduce apoptosis during *S. pneumoniae* infection [100].

Asides from fatty acids, a study proved that the antibacterial activity of flaxseed oil is attributed to cyclolinopeptides, a type of hydrophobic peptides present in flaxseed oil. 1-Mso cyclolinopeptides B and 1-Mso, 3-Mso-cyclolinopeptides F isolated from flaxseed oil exhibited significant antibacterial activity against *Listeria monocytogenes* through destruction of the bacterial cell membrane [101].

The antimicrobial activity of flaxseed oil was tested in vitro against *Staphylococcus aureus* and *Streptococcus agalactiae*, results showed a comparable antibacterial effect of the oil to that of cefoperazone with mean inhibition zones diameter of (17.83 mm and 19.66 mm, respectively for the oil) compared to (17.33 and 18.33, respectively for cefoperazone. On the other hand, a superior antibacterial activity against *Enterococcus faecalis*, *Micrococcus luteus* and *Candida albicans* was exhibited by flaxseed oil compared to cefoperazone with mean inhibition zones diameter of 14.66, 15.83, and 10.33 mm, respectively, for the oil compared to 5.66, 7.66, and 4.33, respectively, for cefoperazone [9].

Another study reported the effect of in vivo supplementation with flaxseed oil in infected sheep with *Fasciola hepatica*. The control group showed increase in body weight and fecal egg count (*P* < 0.05); whereas, flaxseed oil group showed lower number (71.2 ± 26.5) and size of flukes (*P* < 0.01) at necropsy. Furthermore, this group showed higher levels of white blood cells and lymphocytes (*P* < 0.01) as well as red blood cells and hematocrit values (*P* < 0.01). On the other hand, the control group experienced severe hypoalbuminemia and hypoproteinemia with the highest fluke burden (123.0 ± 35.2), together with the highest IgG1 titer (*P* < 0.01) [102].

#### Antioxidant activity

During metabolic reactions, reactive oxygen species, and nitrogen species (ROS/RNS) are continuously produced inside our bodies. These products are crucial for the immune system, energy supply, detoxification, and chemical signaling. Damage to cellular components like DNA, lipids, and proteins results from excessive production of these species which formed due to exposure to external oxidants or disruption in the body's natural antioxidant mechanisms like catalase, glutathione peroxidase, and superoxide dismutase. The increased risk of several degenerative diseases, including cancer, cardiovascular disease, is widely known to be linked to the generation of these free radicals [13].

The value of herbal medicines in clinical context has drawn a lot of attention recently with an increasing interest in their efficacy in lowering free radical-induced tissue damage as many synthetic antioxidants showed negative effects [103]. Generally, antioxidants can be divided into two categories, primary (chain-breaking) antioxidants and secondary (preventive) antioxidants [104]. While secondary antioxidants may function by binding metal ions that can catalyze oxidative processes, absorbing UV radiation, blocking enzymes, or dissolving hydroperoxides, primary antioxidants typically function by giving a hydrogen atom [105].

Most in vitro experiments focused on the antioxidant properties of whole flaxseed rather than its oil. Only one study assessed the in vitro antioxidant activity of flaxseed oil using 2,2-diphenyl-1-picryl-hydrazyl-hydrate (DPPH) and H_2_O_2_ radical scavenging methods. The study showed that flaxseed oil exerted stronger antioxidant activity than ascorbic acid [106].

On the other hand, more attention was drawn towards the indirect protective effect of flaxseed oil on the oxidative stress affecting different body organs. It was proved that daily consumption of 3 ml/kg flaxseed oil for 3 weeks significantly decreased physiological antioxidant enzymes level namely, thiobarbituric acid reactive substances, glutathione, and catalase, found in the liver, kidney and heart induced by streptozotocin in diabetic rats by (2.4, 1.7, 1.6), (2.3, 1.2, 3.5), (97.5, 1.6, 2.9) folds, respectively, indicating for a potential protective effect of the oil against streptozotocin oxidative damage [106]. These results were in agreement with another study that reported improvement in advanced oxidation protein products, malondialdehyde (MDA) and 8-hydroxyguanosine oxidation stress parameters in flaxseed oil-fed diabetic rats [107]. Furthermore, it was found that the daily intake of 15% diet weight flaxseed oil for 10 days before and 4 days after chemotherapy treatment by Cisplatin (6 mg/kg body weight as single dose) significantly attenuated Cisplatin-induced changes in enzymatic and nonenzymatic antioxidant parameters in intestinal homogenates of experimental rats. Whether administration of flaxseed oil with chemotherapeutics can lower its cellular toxicity has yet to be fully elucidated and should add to its health benefits for cancer treatment. Lipid peroxidation was significantly improved by 74%, while superoxide dismutase (SOD) and glutathione peroxidase levels were significantly improved by 48% and 38%, respectively. It was suggested that flaxseed oil exerted this ameliorating effect by two possible mechanisms. First, it increases the levels of antioxidant enzymes in the intestinal mucosal cells, resulting in enhanced defense against oxygen free radicals. Second, ω-3 PUFAs of flaxseed oil may have replaced polyunsaturated fatty acid components of the oxygen free radicals-damaged membranes thereby increasing membrane integrity. It is worth noting that a marked increase in the antioxidant enzyme activities, SOD and catalase, 21% and 36%, respectively, was reported when rats were fed flaxseed oil diet alone without Cisplatin administration [108].

DPPH, ABTS, FRAP and ORAC in vitro assays were conducted to evaluate the free radical scavenging capacity of polar fraction, non-polar fractions, whole oil of flaxseed. It was found that the polar fraction of oil extracted from 160 °C-roasted seeds was better than other oils in terms of DPPH, ABTS, FRAP, and ORAC assays (125, 199, and 122 μmol TE/ 100 g), while the whole oil of cold pressed flaxseed had the highest values of DPPH assay (179 μmol TE/100 g). Correlation between the quantity of flaxseed antioxidant compounds and its free radical scavenging capacity showed that γ-tocopherol had a significant positive correlation with DPPH (polar fraction and whole oil), ABTS, FRAP, and ORAC, whereas phytosterols (except for stigmasterol) were significantly correlated with DPPH (polar fraction and whole oil), ABTS, FRAP and ORAC. A significant positive correlation was also found between flavonoids and antioxidant activity as determined using ABTS assay. Results indicated that appropriate roasting might be beneficial in improving the oxidative stability and free radical scavenging capacity of pressed flaxseed oil by increasing the content of these bioactive compounds [12]. Whether seed roasting could generate melanoidin compounds of known antioxidant action and to account for the improved efficacy in oil extracted from roasted seed is not examined and profiling of processed oil should aid confirm such hypothesis.

Likewise, another parameter to affect flaxseed oil composition is seed germination. A study compared the antioxidant activity of germinated flaxseed with ungerminated ones revealing that after 4 days, ungerminated seeds showed higher antioxidant activity than that of germinated ones. Although, antioxidant activity of germinated seeds oil ranged from 40.14 to 52.48% during germination days, it was observed that it significantly decreased from day 1 up to day 3 then further increased at day 4 [109]. We suggest as future work for the analysis of flaxseed oil from germinated versus ungerminated seeds to provide a conclusive evidence for the role of germination in flaxseed oil antioxidant effect as the concentration of compounds responsible for the antioxidant activity might vary at different stages of germination. Figure 1 summarizes optimum conditions for improved antioxidant capacity of flaxseed oil components.

#### Other health effects

Flaxseed oil showed a protective effect against gastric ulcers induced by ethanol in rats. Oral pre-treatment of rats (5 ml/kg) with flaxseed oil resulted in a significant reduction in the number (*P* < 0.001) and length of ulcers (*P* < 0.001) relative to control animals pre-treated with an equivalent amount of corn oil. Flaxseed at a dose of 5.0 ml/kg was more prominent than that achieved by ranitidine (50 mg/kg) [110], and can be used in combination with antacids drug to lower their side effects especially being consumed typically over long period of time.

Moreover, Flaxseed oil has been reported for its remedial effect on dry eye syndrome**,** oral flaxseed oil capsules of 1 or 2 g/day reduced ocular surface inflammation and improved the symptoms of keratoconjunctivitis sicca in Sjögren's syndrome patients [111]. The safety and efficacy of a nano-emulsion artificial tear consisting of carboxymethylcellulose, glycerin, flaxseed oil, and trehalose as osmoprotectant was compared with a commercial artificial tear containing the same ingredients except for trehalose and flaxseed oil. Results revealed a significant improvement using the novel nano-emulsion tear formulation for combined ocular staining, corneal staining, and conjunctival staining [112].

## Flaxseed synergistic effects with other oils and drugs

Considering that there were several reports in literature on flaxseed oil effects in combination with other oils or drugs, a highlight of the main finding of these studies is presented in that section. A study reported the effect of extruded flaxseed and soybean on Holstein dairy cows’ milk yield, fatty acid composition, and first-service conception rate. A test group was fed a basal diet with a supplementation of 650 g/kg of extruded flaxseed and 150 g/kg of extruded soybean at a rate of 100 g/kg compared to the control group which was fed on basal diet only. Results showed that daily milk yield and solid not fat yield increased by 3.26% and 0.88%, respectively, in the test group in comparison to the control. Moreover, a significant decrease in percentage milk fat by 1.4% was observed in the test group compared to the control group. With regard to milk composition, milk profile of the supplemented group with of extruded flaxseed and soybean showed a decrease in saturated fatty acids and an increase in monounsaturated and polyunsaturated fatty acids. Although the change in the milk fat profile, the first-service conception rate was not affected [8]. Another study reported that the milk yield was higher in cows fed a diet supplemented with flaxseed oil a resulting in 8.97% increase compared with the control diet, The DFSO displayed decreased short-chain fatty acids, 3.33%, when compared to CD for buffalo milk (6.3%) [113].

A study reported the effects of flaxseed oil and olive oil on inflammatory markers and wound healing. The study included 112 patients with burns were divided into four groups (flaxseed oil group, olive oil group, combination of olive oil and flaxseed group, and control group) receiving 30 g of both oils for three weeks. Results showed that the strongest decrease in the level of serum high-sensitivity C-reactive protein was observed in case of mixture of olive oil and flaxseed (− 21.38 ± 44.41) (− 132.79 ± 165.36), while the lowest reduction was reported in the control group (− 36.36 ± 79.03) (141.08 ± 262.36) [114].

Another synergistic effect was reported where a phenolic extract obtained from flaxseed oil in combination with Doxorubicin induced cytotoxicity to MCF-7 cells as well as apoptosis, mitochondrial membrane depolarization and cell cycle modification. This combination may suggest future lowering of doxorubicin dose in cancer patients [82].

A synergistic hepatoprotective effect of flaxseed oil and sesame seed oil was reported against carbon tetrachloride induced liver damage in rats. The ω-3 and ω-6 FAs from both oils showed significant synergistic antioxidant and hepatoprotective effects. These results were manifested by increased in vivo antioxidant enzymes activities such as catalase, superoxide dismutase, and peroxidase enzymes [115]. Moreover, flaxseed oil also showed a synergistic antioxidant effect when supplemented in combination with coconut oil [116].

Administration of ALA rich oils in combination with Angiotensin-converting enzyme inhibitors decreased blood pressure to 7-week-old spontaneously hypertensive rats. The systolic blood pressure was reduced significantly in rats which administrated ALA with ACE-I in comparison to the systolic pressure level before administration [117].

Flaxseed extract in combination with spearmint oil was reported to improve endocrine and histomorphology of ovary of experimental polycystic ovary syndrome. Primary, pre-antral and antral follicles showed a significant increase in the treatment group. On the other hand, a decrease in both the number of cystic follicles as well as thickness of theca was observed while an increase in granulosa layer thickness in comparison to the untreated group (*P* < 0.05) [118].

## Future perspectives

Although various studies are available on flaxseed oil health and therapeutic benefits, more investigations are still needed to reveal the effect of different oil extraction methods on the chemical composition and hence its several health benefit only assessed in case of antioxidant action. Moreover, more research studies are required to report on the chemical variation in flaxseed oil composition in different flaxseed varieties, and their pharmacological activities in animal models and human trials. Indeed, more in vivo studies as well as controlled clinical trials are required to provide better evidence for less explored effects such as its neuroprotective effects for future therapy for control and management of neurodegenerative diseases particularly Alzheimer’s disease. Future research should also focus on the synergistic effect of flaxseed oil combined with other natural extracts rich in PUFAs. Flaxseed oil presents a promising agent for pharmaceutical and nutraceutical industries in improving human health and this requires more data about its stability, possibility of new formulations, and delivery systems.

## Conclusions

Flaxseed oil, the major product of flaxseed, is highly recognized for its several therapeutic benefits which are mainly attributed to its unique chemical composition. Flaxseed oil presents one of the rich sources of omega-3 and omega-6 fatty acids where α-linolenic and linoleic fatty acids are the main components, respectively. Studies revealed that the consumption of high flaxseed oil supplements in diet exerted a promising effect in treatment as well as prevention of various diseases. Reduction in the risk of heart diseases like arrhythmia and sudden cardiac death is interlinked with the high percent of EPA and DHA levels in flaxseed oil. These oil components are responsible for the significant lowering in plasma cholesterol and blood pressure in dyslipidemic patient. Moreover, high EPA levels in flaxseed oil contributed to the improvement of many CNS depression symptoms such as behavioral despair and anhedonia. α-Linolenic fatty acids in flaxseed oil has anti-inflammatory effects due to decreasing the production of inflammatory cytokines. The richness of flaxseed oil in PUFAs resulted in a prominent anti-inflammatory, immunoregulatory and antibacterial effects. Additionally, flaxseed oil dietary consumption improved osteoporotic status by improvement of femur bone mineral density, bone strength, and fatty acids composition mostly attributed to its lipophilic components that are PUFA and to less extent to lignans.

**Fig. 5:**
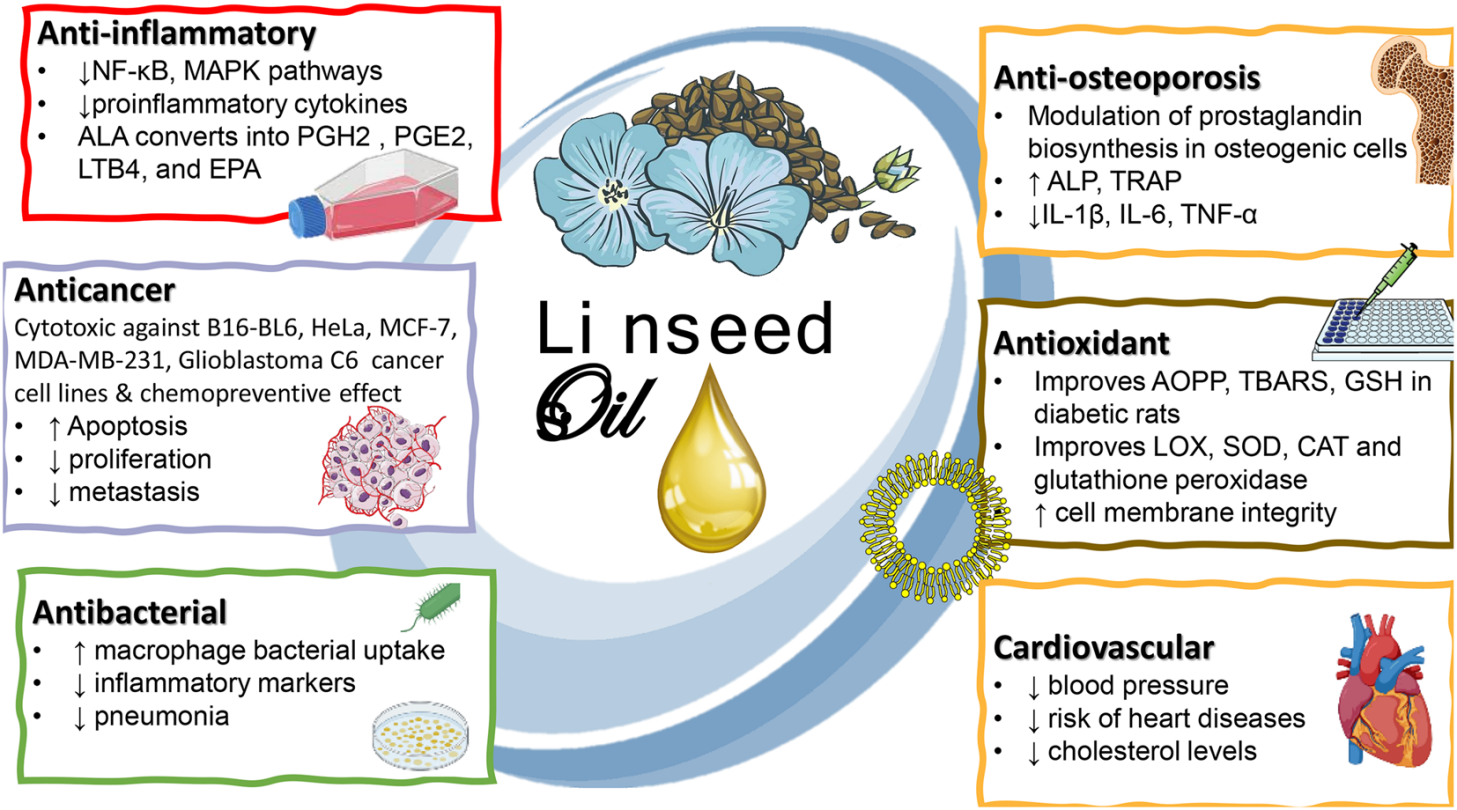
Flaxseed oil therapeutic and health uses and the underlying mechanisms of action

Other components to contribute to flaxseed oil effects such as antioxidant and anticancer effects asides from PUFAs include tocopherols, beta-carotene, phytosterols and polyphenols. A summary of flaxseed oil effects on the different ailments and the underlying action mechanism is presented in Fig. 5.

## Data Availability

Available if needed.
